# Effects of a 6-Month Educational Program on Blood Pressure in Pre-Frail and Frail Older Adults: A Randomized Controlled Trial

**DOI:** 10.3390/healthcare14060756

**Published:** 2026-03-18

**Authors:** Laura Ávila-Cabeza-de-Vaca, Alba Mier-Perulero, Lacrimioara Tania Tirnovan, Manuel Costilla, Cristina Casals, Andrea González-Mariscal, Juan Corral-Pérez

**Affiliations:** 1 ExPhy Research Group, Department of Physical Education, Instituto de Investigación e Innovación Biomédica de Cádiz (INiBICA), Universidad de Cádiz, 11003 Cádiz, Spain; laura.avila@uca.es (L.Á.-C.-d.-V.); alba.mierperulero@alum.uca.es (A.M.-P.); manueljesus.costilla@uca.es (M.C.); cristina.casals@uca.es (C.C.); juan.corral@uca.es (J.C.-P.); 2 Hospital Regional Universitario de Málaga, 29010 Málaga, Spain; taniatirnovan@gmail.com; 3 School of Health Sciences, International University of La Rioja, Av. de la Paz, 137, 26006 Logroño, Spain

**Keywords:** health education, frailness, blood pressure determination, health promotion, nutritional education, exercise, gerontology, healthy aging, cognition, cardiovascular risk factor

## Abstract

**Background/Objectives**: Maintaining healthy blood pressure (BP) is essential in the frailty phenotype to prevent cardiovascular events. This study examined the effects of a 6-month educational program on BP in community-dwelling pre-frail or frail older adults. **Methods**: In this multicenter randomized controlled trial, 210 community-dwelling older adults (145 women; 74 ± 6 years) meeting at least one of Fried’s frailty criteria were assigned to a control group (usual care; n = 95) or an intervention group (educational program; n = 115). The 6-month intervention consisted of an educational program that provided recommendations on physical activity, nutrition, cognition, and psychosocial well-being, delivered through four group sessions and six telephone calls. Systolic BP and diastolic BP were measured at baseline and after 6 months, and categorized as normal, high-normal, or hypertensive (systolic: <130, 130–139, ≥140 mmHg; diastolic: <85, 85–89, ≥90 mmHg, respectively). **Results**: A significant reduction in systolic BP was observed within the intervention group (Z = −2.84, *p* < 0.01, r = 0.27), increasing the proportion of participants with normal systolic BP by 16% and reducing the proportion of participants with high-normal and hypertensive systolic BP by 8% each (χ^2^ (3) = 9.21, *p* = 0.03). No significant changes were observed in the control group (*p* > 0.05), nor were there significant effects for diastolic BP in any study group (*p* > 0.05). **Conclusions**: These findings suggest that this educational program may be a feasible complementary non-pharmacological strategy to improve systolic BP, a key cardiovascular risk factor, in community-dwelling pre-frail and frail older adults.

## 1. Introduction

Frailty is a condition characterized by a progressive decrease in physiological reserves and a reduced resistance to stressors, leading to increased vulnerability to adverse health outcomes, including falls, hospitalization, disability, and premature death [1]. To identify this condition, Fried proposed an operational definition of frailty based on the presence of three or more of the following criteria: unintentional weight loss, self-reported exhaustion, low physical activity, slow walking speed, and weakness (low grip strength) [1]. Individuals meeting one or two criteria are classified as pre-frail, while those meeting three or more are frail [1]. Currently, the prevalence of this condition is rising; in Spain specifically, it affects 42% (pre-frail) and 8% (frail) of older adults aged ≥65 years [2]. The clinical significance of this high prevalence is exacerbated by the potent association between frailty and chronic systemic pathologies.

Frailty serves as a strong, independent risk factor for cardiovascular dysfunction [3,4]. Within this context, persistently elevated blood pressure (BP) and its clinical diagnosis of hypertension (BP ≥ 140/90 mmHg) represent the most prevalent chronic conditions in older adults and a leading contributor to cardiovascular disease, stroke, and premature mortality [5]. Its prevalence is particularly high among pre-frail (73%) and frail individuals (83%) compared to robust peers (52%) [6]. Current evidence suggests a bidirectional relationship between these entities [7]; while chronic hypertension accelerates vascular decline, the physiological hallmarks of frailty, such as increased arterial stiffness, endothelial dysfunction, and chronic low-grade inflammation, directly impair autonomic control and BP regulation [8,9]. Crucially, the coexistence of frailty and hypertension has been linked to deteriorated clinical outcomes, including disability, depression, and impaired physical and cognitive function [10,11]. Therefore, effective management of BP and hypertension in this vulnerable population is critical. Such management is essential not only to reduce morbidity and preserve independence but also to mitigate the compounded risks of this dual burden, ultimately improving quality of life and long-term health.

Traditionally, interventions for controlling BP in older adults have emphasized pharmacological management or single-component strategies [12,13]. However, non-pharmacological approaches are gaining increasing importance as adjunct interventions for a more comprehensive treatment of this population. Specifically, resource-light, multicomponent educational interventions have emerged as a novel strategy to mitigate this dual burden while fostering patient autonomy and avoiding the risks of polypharmacy. A systematic review of nurse-led educational programs, combining elements such as personalized education, counseling, follow-up support, and healthy lifestyle promotion, reported reductions in BP in older adults aged 60 and above [14]. Despite these interesting results, the presence of a frailty phenotype in the participants was not reported, which limits the applicability of their BP findings to pre-frail or frail older adults. Given the dual burden of frailty and hypertension, a more integrated approach in the frail phenotype population is required. Educational programs that integrate physical, nutritional, cognitive, and social components while promoting self-management and patient engagement have shown benefits in improving physical function, nutrition, and social well-being in community-dwelling pre-frail and frail older adults [15,16,17]. Despite these functional benefits, there is a notable lack of randomized controlled trials targeting BP outcomes specifically in pre-frail and frail populations. Consequently, there remains significant uncertainty regarding the effectiveness of multicomponent educational interventions on cardiovascular outcomes in this vulnerable group.

Therefore, the present study aimed to assess the effect of a 6-month educational program on BP among community-dwelling pre-frail or frail older adults.

## 2. Materials and Methods

### 2.1. Study Design and Ethical Approval

This 12-month multicenter, randomized controlled trial (FRAGSALUD project) was conducted between March 2022 and September 2023 in 14 healthcare centers in Andalusia, Spain. Although the overarching project included a long-term follow-up, the present analysis focuses specifically on the active intervention period (baseline to 6 months) to evaluate the immediate efficacy of the educational program on cardiovascular and behavioral outcomes, deliberately isolating the impact of the active stimulus from potential long-term adherence decay. The trial was registered at ClinicalTrials.gov (identifier: NCT05610605) and received approval from the Provincial Research Ethics Committee of Málaga (reference code FRAGSALUD, dated 31 January 2019). All procedures complied with the Declaration of Helsinki and Good Clinical Practice Guidelines [18]. Prior to participation, all individuals received comprehensive study information and provided written informed consent. The manuscript was prepared following the CONSORT 2025 guidelines for reporting randomized controlled trials [19].

### 2.2. Sample Size and Randomization

The initial sample size was calculated to detect a medium effect size (Cohen’s d = 0.50) in the primary outcome (Fried’s frailty status), assuming a two-sided alpha level of 0.05 and 80% statistical power. This required 85 participants per group. To account for a projected 15% dropout rate, the target recruitment was set at 196 participants (98 per group). For the analysis of BP, a sensitivity analysis was performed to account for the use of non-parametric procedures (Mann–Whitney U and Wilcoxon signed-rank tests). Given the asymptotic relative efficiency of these tests (typically 0.864 relative to parametric equivalents), the final target of N = 98 per group provided a post-adjustment power of 81.4% to detect the original medium effect size (d = 0.50, equivalent to r = 0.24). Furthermore, sensitivity analysis indicated that the final sample was sufficient to detect effect sizes as small as r = 0.21 with alpha = 0.05 and 80% power. These metrics ensure that the study is robustly powered to identify clinically meaningful differences in BP despite the possible non-normal distribution of the data.

Participants were randomly assigned in a parallel-group design using a computer-generated sequence created with the software Randomization (http://www.randomization.com). Block randomization with a fixed block size was employed to ensure balanced allocation between groups. The randomization sequence was generated prior to participant enrollment. Due to the nature of the educational intervention, the study followed an open-label design with no masking; therefore, neither participants nor investigators were blinded to group allocation.

### 2.3. Participants

Community-dwelling adults aged 65 years or older were eligible if they met at least one of Fried’s frailty criteria [1] and had the full cognitive capacity to participate voluntarily. Individuals classified as robust (no frailty criteria met) or institutionalized were excluded.

Frailty status was assessed using Fried’s criteria [1], which comprise five domains:

- Unintentional weight loss: ≥4.5 kg or ≥5% body weight unintentional decrease in the preceding year.
- Self-reported exhaustion/fatigue: Feeling exhausted or fatigued for ≥3 days in the week before assessment.
- Low weekly physical activity expenditure: <383 kcal/week for men or <270 kcal/week for women, determined using the validated Spanish short version of the Minnesota Leisure Time Activity Questionnaire (VREM), which has shown excellent reproducibility (ICC = 0.95) [20].
- Low gait speed: For men, walking 4.57 m in ≥7 s if height ≤ 173 cm, or ≥6 s if height > 173 cm. For women, ≥7 s if height ≤ 159 cm, or ≥6 s if height > 159 cm.
- Low handgrip strength: Measured with a hand dynamometer (Takei T.K.K.5401 GRIP-D handgrip dynamometer, Takei Scientific Instruments Co., Ltd., Tokyo, Japan). Participants were classified as having low handgrip strength based on cut-off points that accounted for both sex and body mass index (BMI). For men, the cut-off points were ≤24 kg for BMI ≤ 24 kg/m^2^, ≤30 kg for BMI 24–28 kg/m^2^, and ≤32 kg for BMI > 28 kg/m^2^. For women, the corresponding cut-offs were ≤17 kg for BMI ≤ 23 kg/m^2^, ≤17.3 kg for BMI 23–26 kg/m^2^, ≤18 kg for BMI 26–29 kg/m^2^, and ≤21 kg for BMI > 29 kg/m^2^ [1]. Body height was assessed in a standing position based on the Frankfort plane, after normal expiration, using a stadiometer (TANITA-LEICESTER HR-001, Tanita Corp., Tokyo, Japan). Body mass was measured with an Omron BF-400 device (Omron Medizintechnik, Mannheim, Germany), with participants wearing minimal clothing. BMI was subsequently calculated as body mass (kg) divided by height squared (m^2^).

Participants meeting one or two criteria were classified as pre-frail, and those meeting three or more were classified as frail.

### 2.4. Intervention

The 6-month educational intervention was delivered to the intervention group by a multidisciplinary team of nurses, nutritionists, and physical educators. To ensure intervention fidelity, all sessions were delivered by professionals with specific expertise in their respective content areas. Prior to the start of the study, the multidisciplinary team held structured coordination meetings to standardize the content, objectives, and delivery procedures of each session, thereby ensuring consistency across groups and facilitators. During the first month, participants attended four weekly group sessions of 45–60 min each. The first session focused on frailty awareness, including health implications, age-related progression, and prevention strategies. The second session addressed exercise guidelines, with recommendations for strength and aerobic training, home-based examples, and encouragement of regular activity. The third session focused on preventing protein-calorie malnutrition and dehydration, providing practical advice and adaptations tailored to personal situations. The fourth session promoted cognitive and social engagement through cognitive exercises (e.g., crosswords, sudoku, crocheting) and activities to foster social participation. Each session was limited to a maximum of 15 participants and conducted in accessible community venues, such as neighborhood associations, healthcare centers, or university facilities. Adherence was prospectively monitored using attendance logs, and full participation in all sessions was predefined as the adherence criterion. Full participation in all four sessions was predefined as the minimum adherence criterion. Therefore, missed sessions were systematically rescheduled in alternative groups to ensure completion of all modules. Following the initial month, participants received six follow-up telephone calls: two biweekly calls during the second month and one monthly call from the third to the sixth month. These calls reinforced adherence, addressed barriers, and suggested adaptations when needed. The control group continued to receive standard healthcare without additional educational sessions or follow-up calls. Details of the full intervention are provided in a previously published study [15].

### 2.5. Blood Pressure

The BP was measured in the healthcare center by trained staff using an automatic oscillometric monitor (Omron M2+ HEM-7146-E, Omron Healthcare, Kyoto, Japan). After a minimum 5 min rest in a seated position with the back supported, feet flat on the floor, and legs uncrossed, the cuff was placed on the bare upper left arm, unless a medical indication required the use of the right arm, with its lower edge positioned approximately 2–3 cm above the antecubital fossa. The arm was supported at heart level throughout the measurement. Two readings were taken one minute apart, and the mean of these two values was used for subsequent analysis.

According to the European Society of Cardiology and the European Society of Hypertension guidelines [21], BP was then classified into clinical ranges based on mean values: Systolic measurements were categorized as normal (<130 mmHg), high-normal (130–139 mmHg), or hypertensive (≥140 mmHg), and Diastolic measurements were categorized as normal (<85 mmHg), high-normal (85–89 mmHg), or hypertensive (≥90 mmHg).

### 2.6. Pharmacological Burden

The total daily medication burden was assessed through structured clinical interviews at baseline and the 6-month follow-up. Specifically, participants were asked to count and report the total number of distinct medications they were currently taking on a daily basis. The pharmacological burden was defined strictly as this overall numerical count of daily drugs consumed by each participant. This quantitative approach was used to monitor potential fluctuations in the overall pharmacological regimen that could influence blood pressure outcomes throughout the intervention period.

### 2.7. Statistical Analysis

In accordance with CONSORT 2025 reporting standards [19], a Complete Case Analysis was conducted without data imputation. Normality of the data distribution was assessed using the Kolmogorov–Smirnov test, and the homogeneity of variances was examined with Levene’s test. Continuous variables are presented as means ± standard deviations when data is normally distributed, or as medians with interquartile ranges (IQRs) when data is non-normally distributed. Categorical variables are presented as counts and percentages.

Given the non-normal distribution of the outcome variables (BP and total daily medications), as evidenced by significant skewness in the data, non-parametric statistical methods were employed. Accordingly, within-group comparisons between baseline and 6-month assessments for systolic and diastolic BP, and total daily medications were performed using the Wilcoxon signed-rank test. Effect sizes were calculated as r = Z/(√*N*), where Z is the standardized test statistic derived from the Wilcoxon test, and *N* is the number of participants included in the analysis. Effect sizes were interpreted according to Cohen’s criteria: small (r = 0.1), medium (r = 0.3), and large (r = 0.5) [22]. Between-group differences in post-intervention outcomes were analyzed using the Mann–Whitney *U* test.

Within-group changes in categorical outcomes (systolic and diastolic BP categories: normal, high-normal, and hypertension) were assessed using the McNemar–Bowker test of symmetry. This test is an extension of the McNemar test for paired categorical data with more than two categories. In our study, the three BP categories at baseline were cross-tabulated with the same three categories at post-intervention, resulting in 3 × 3 contingency tables. The test was applied separately for the intervention and control groups to determine whether the intervention or the absence of the intervention produced significant shifts in the BP category. Between-group differences in post-intervention categorical outcomes were analyzed using the chi-square test.

Statistical significance was set at *p* < 0.05. All analyses were performed using IBM SPSS Statistics 25 software (SPSS Inc., Chicago, IL, USA). Figures illustrating the distribution of systolic and diastolic BP categories were created using GraphPad Prism 6 software (GraphPad Software, Boston, MA, USA).

## 3. Results

**Figure 1:**
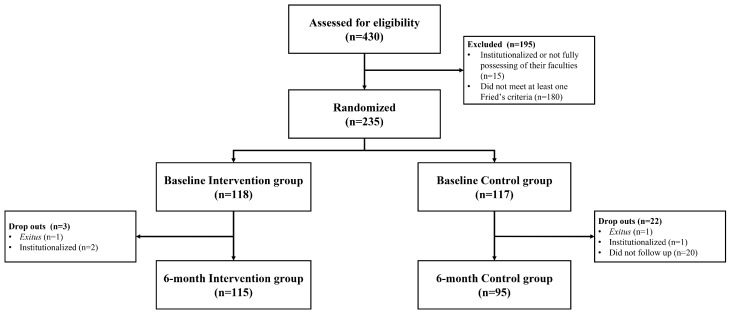
Flowchart of the 6-month FRAGSALUD study.

Out of 235 participants initially randomized in the study, 210 completed the 6-month evaluation. A total of 25 participants were lost to follow-up, comprising 22 participants (18.8%) from the control group and 3 participants (2.5%) from the intervention group. Consequently, the 6-month analysis included 115 participants in the intervention group and 95 participants in the control group (Figure 1). To rigorously rule out attrition bias resulting from this differential dropout rate, a missing value analysis was performed. Little’s Missing Completely at Random (MCAR) test showed no evidence of systematic bias (χ^2^ (2) = 1.11, *p* = 0.58), confirming that data were missing completely at random. Consequently, a per-protocol analysis was deemed entirely appropriate. Baseline characteristics of the participants, including age, anthropometric measurements, BP, and sex, are summarized and presented in Table 1, with values reported for the total sample, as well as for the control and intervention groups.

### 3.1. Systolic BP

Baseline systolic BP did not differ significantly between the intervention and control groups (U = 4870.50, z = −1.37, *p* = 0.17). In the intervention group, systolic BP decreased significantly after the 6-month educational program, showing a small-to-medium effect size (Baseline Median = 130 mmHg, IQR = 120–140; Post Median = 130 mmHg, IQR = 115–140; z = −2.84, *p* < 0.01, r = 0.27). Although the median remained unchanged, the significant result reflects a shift in the distribution toward lower values, particularly in the upper tail. Consistent with this pattern, the maximum systolic BP decreased from 190 to 160 mmHg and the 95th percentile from 168.4 to 152.2 mmHg. In contrast, no significant change was observed in the control group (Baseline Median = 130 mmHg, IQR = 120–140; Post Median = 130 mmHg, IQR = 120–135, z = −0.42, *p* = 0.67, r = 0.04). Post-intervention comparisons revealed no significant difference between groups (U = 5239.50, z = −0.51, *p* = 0.61). To maximize clinical transparency, systolic BP values are also presented as means and standard deviations, including subgroup distributions, in Supplementary Table S1. Furthermore, visual distribution histograms relative to the hypertension diagnostic targets are provided in Supplementary Figure S1.

**Figure 2:**
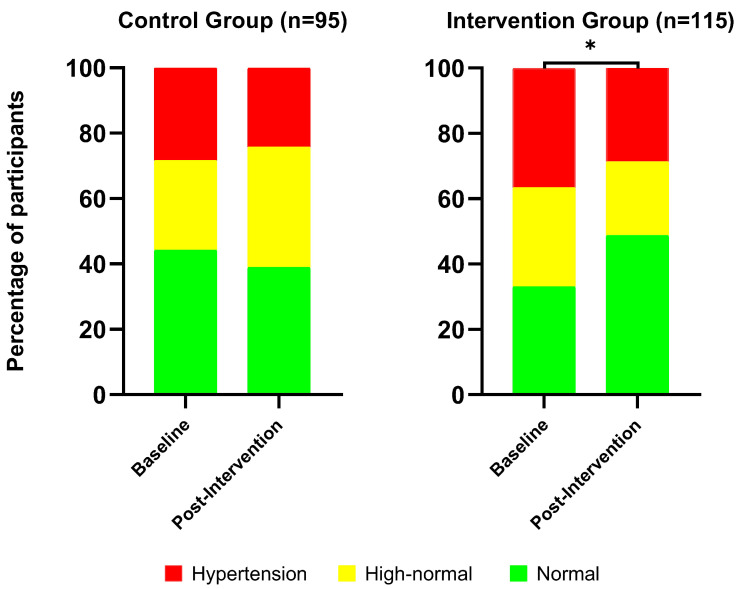
Distribution of systolic BP categories (normal tension, high-normal tension, and hypertension) at baseline and post-intervention for both the control and intervention groups. Asterisk (*) indicates a significant difference (*p* < 0.05) between the distribution of systolic BP categories.

Regarding categorical distribution, baseline analysis showed no significant association between group and systolic BP category (χ^2^ (2) = 2.91, *p* = 0.23). Post-intervention analysis revealed a trend toward association (χ^2^ (2) = 5.14, *p* = 0.08), with the control group showing a higher-than-expected proportion of participants in the high-normal category and the intervention group showing a higher-than-expected proportion in the normal category. Within-group changes in systolic BP categories were significant for the intervention group (χ^2^ (3) = 9.21, *p* = 0.03), increasing the proportion of participants with normal systolic BP (+16%) and reducing the prevalence of high-normal (−8%) and hypertensive (−8%) systolic BP categories, but not for the control group (χ^2^ (3) = 4.25, *p* = 0.24) after 6 months. The distribution of systolic BP categories (normal, high-normal, and hypertensive) at baseline and post-intervention for both the control and intervention groups is shown in Figure 2.

### 3.2. Diastolic BP

Baseline diastolic BP did not differ significantly between groups (U = 4932.50, z = −1.24, *p* = 0.22). Within the intervention group, diastolic BP did not change significantly (Baseline Median = 78 mmHg, IQR = 70–80; Post Median = 75 mmHg, IQR = 70–80; z = −0.32, *p* = 0.75, r = 0.03). Similarly, no significant change was observed in the control group (Baseline Median = 75 mmHg, IQR = 70–80; Post Median = 75 mmHg, IQR = 70–80; z = −0.93, *p* = 0.35, r = 0.09). Post-intervention comparisons revealed no significant difference between groups (U = 5219.50, z = −0.56, *p* = 0.56). To maximize clinical transparency, diastolic BP values are also presented as means and standard deviations, including subgroup distributions, in Supplementary Table S1. Furthermore, visual distribution histograms relative to the hypertension diagnostic targets are provided in Supplementary Figure S2.

**Figure 3:**
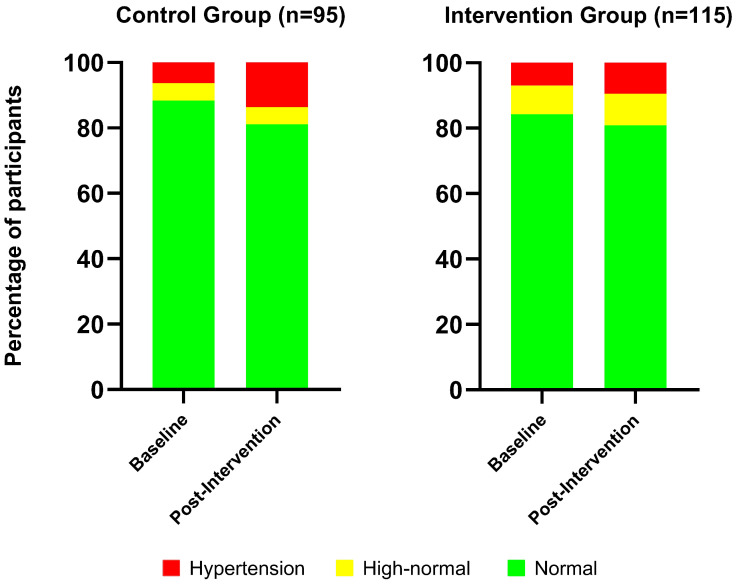
Distribution of diastolic BP categories (normal tension, high-normal tension, and hypertension) at baseline and post-intervention for both the control and intervention groups.

Analysis of categorical distribution showed no significant association between group and diastolic BP category at baseline (χ^2^ (2) = 2.91, *p* = 0.23) or post-intervention (χ^2^ (2) = 2.04, *p* = 0.36). Likewise, within-group changes in diastolic BP categories were not significant in either the intervention (χ^2^ (3) = 3.87, *p* = 0.28) or control group (χ^2^ (3) = 4.74, *p* = 0.19). The distribution of diastolic BP categories (normal, high-normal, and hypertensive) at baseline and post-intervention for both the control and intervention groups is shown in Figure 3.

### 3.3. Pharmacological Burden

To assess whether the observed BP changes were influenced by pharmacological adjustments, the total daily medication burden was analyzed. At baseline, there were significant differences between groups (U = 4310.50, z = −2.07, *p* = 0.038; *r* = 0.14; Median = 4 [2–7] for intervention and 5 [3–8] for control group). No significant within-group changes in the number of medications were observed after 6 months for either the intervention group (Baseline Median = 4, IQR = 2–7; Post Median = 4, IQR = 2–6; z = −1.46, *p* = 0.14) or the control group (Baseline Median = 5, IQR = 3–8; Post Median = 5, IQR = 2.5–8; z = −1.91, *p* = 0.05).

## 4. Discussion

This randomized controlled trial evaluated the impact of a 6-month educational program on BP in community-dwelling pre-frail and frail older adults. The main finding was that the educational program achieved a significant intragroup reduction in systolic BP levels, characterized by an attenuation of extreme values and a decrease in the proportion of participants with systolic hypertension. Nevertheless, no significant effects were observed for diastolic BP. These changes are particularly noteworthy, as the intervention focused on broader frailty phenotype (physical activity, nutrition, cognitive, and social engagement) rather than specifically targeting hypertension management. This highlights the potential for educational interventions primarily focused on frailty to yield secondary, clinically meaningful cardiovascular benefits, addressing the dual burden of these conditions in the aging population.

The observed within-group reduction in systolic BP may have important clinical implications for pre-frail and frail older adults. Age-related increases in BP have been shown to increase the risk of stroke, heart failure, and premature mortality [23]. Previous studies provided evidence on the effectiveness of multicomponent interventions on BP control in general older adults without characterization of frailty [8]. These successful programs often share key features, such as combining health education, motivational meetings with personalized action plans, dietary advice, and encouragement of physical activity to reduce systolic BP [24], promoting self-management with caregiver support [25], or integrating discharge education with coordinated primary care follow-up [26]. In contrast, interventions focused on medication reminders [12] or education about patients’ understanding of hypertension [13] improved medication adherence without achieving systolic BP reductions. Collectively, these studies suggest that multi-component programs could be more effective than unidimensional strategies.

Our findings suggest that educational strategies can promote BP reductions in pre-frail and frail older adults. This finding is particularly relevant because frailty, although potentially reversible, usually follows a trajectory of accelerated physiological decline [27]. In such a vulnerable context, where physiological reserves are already compromised, even modest decreases in systolic BP may yield meaningful clinical benefits [28], potentially improving quality of life and independence [29,30]. Particularly noteworthy is the intervention’s impact on the upper extremes of the BP distribution: the maximum systolic value dropped from 190 to 160 mmHg, and the 95th percentile was reduced from 168.4 to 152.2 mmHg. These improvements were attained through a resource-light format (four group sessions and six telephone calls) covering frailty awareness, physical activity, nutrition, cognition, and social engagement, despite not focusing on BP control. This highlights the feasibility and potential value of integrated frailty care; by addressing the underlying phenotype, we can simultaneously mitigate secondary cardiovascular stressors. Crucially, these improvements were achieved while maintaining a stable pharmacological profile among participants, suggesting that the observed benefits were primarily associated with the educational and lifestyle modifications rather than treatment adjustments. In a population frequently burdened by progressive physiological decline, these improvements may help prevent the need for future escalation of pharmacological treatments. Our observations of a stable medication burden throughout the study support this perspective, as it suggests that lifestyle-focused interventions can mitigate the risks and adverse events associated with increased polypharmacy in frail older adults. Future research should investigate whether these lifestyle shifts can be sustained long-term to maintain cardiovascular stability without further increasing the pharmacological load.

Regarding diastolic BP, no significant changes were found, which is likely explained by a floor effect. Most of the participants in our trial were already within normal diastolic BP ranges at baseline (median 75–78 mmHg), limiting the potential for measurable improvements. This finding is consistent with the physiological trajectory of aging, as diastolic BP tends to stabilize or even decline in later life due to arterial stiffening and reduced vascular compliance [23]. In pre-frail and frail older adults, where vascular rigidity and diminished physiological reserves are common [31], the potential for further reduction in diastolic BP may be physiologically limited, restricting the responsiveness to lifestyle interventions observed in this cohort.

Moreover, studies targeting older adults with relatively well-controlled BP reported improvements in adherence, knowledge, or self-efficacy, but no significant reductions in diastolic BP [12,32,33]. However, when baseline BP in older adults is uncontrolled, different types of interventions, including nurse-led hypertension management programs [24], self-management programs [25], and transitional care strategies [26], have shown parallel reductions in both systolic and diastolic BP. The differences in study sample characteristics compared with our study suggest that baseline BP levels strongly influence the effectiveness of the intervention. Moreover, these comparisons indicate that diastolic BP may be less modifiable in frail older adults who already present values within the normal range.

Beyond absolute values, this study emphasized categorical shifts as a primary clinical indicator. This approach can provide a clinically relevant perspective on cardiovascular risk that is often missing from previous studies [12,24,25]. Previous interventions, such as the Risk Assessment and Management Program and Hong Kong Hypertension Intervention Nurse-led trials [34,35], produced reductions in older adults classified as hypertensive through structured educational programs and follow-up focused on hypertension self-management and lifestyle modification. However, these studies did not specify participants’ frailty phenotype, and did not analyze systolic and diastolic BP separately, despite their different physiological fluctuations with aging [36]. Remarkably, our 6-month educational program substantially increased the proportion of participants achieving normal systolic BP. Maintaining systolic BP within normal ranges is particularly critical in frail older adults. Systolic hypertension can compromise cardiovascular stability, increase the risk of stroke, myocardial infarction, and negatively impact functional independence [30]. By keeping systolic BP in the normal range, interventions like ours can improve cardiovascular health and preserve overall resilience in this vulnerable population.

Regarding diastolic BP categories, no significant shifts were observed in our study. While monitoring diastolic BP remains clinically important for identifying risks like impaired organ perfusion [37], our findings align with the physiological trajectory of aging, suggesting systolic BP is the more modifiable and pressing target in this frail cohort.

Nevertheless, several aspects warrant careful consideration. First, BP was measured at discrete points in time, and continuous or ambulatory monitoring could provide a more comprehensive assessment of BP variability and intervention effects. Second, the lack of significant differences between groups at 6-month limits causal inference, suggesting that these results be interpreted as longitudinal improvements within the intervention group. Third, while we prospectively monitored the overall medication burden (total number of prescribed daily drugs) to account for polypharmacy, we did not track specific dose titrations or changes within distinct antihypertensive medication classes, nor did we objectively measure daily pill adherence using a validated questionnaire. Although the stability of the total medication count throughout the 6-month follow-up suggests that broad pharmacological escalation did not drive the observed hemodynamic improvements, the lack of granular data on specific cardiovascular drug adjustments prevents a definitive exclusion of minor pharmacological confounding. Finally, the study was conducted in a specific geographic and healthcare context (Andalusia, Spain), which may limit the generalizability of the findings. This setting is characterized by a universal public healthcare system that facilitates access to primary care, as well as cultural factors such as strong family support networks, which may positively influence participants’ engagement and adherence.

Despite these limitations, the study has several strengths. The educational intervention was tailored to pre-frail and frail participants and delivered in small groups with structured calls, which likely increased accessibility. BP assessment included both absolute values and categorical classifications (normal, high-normal, hypertensive), providing a clinically meaningful perspective on cardiovascular risk. These design features strengthen the robustness and real-world applicability of the findings, supporting the potential implementation of similar programs in community settings.

## 5. Conclusions

This randomized controlled trial suggests that a 6-month educational program, comprising four group sessions and six telephone follow-ups, integrating physical activity, nutrition, and cognitive-social engagement, can reduce systolic BP in community-dwelling pre-frail and frail older adults. While no significant differences were observed between groups at follow-up, the intervention was associated with an increased proportion of participants achieving normal systolic BP and a reduced prevalence of high-normal and hypertensive systolic BP categories. Furthermore, it effectively reduced extreme hypertensive peaks and the highest values of the systolic blood pressure distribution, whereas diastolic BP remained unaffected.

These findings suggest that non-pharmacological, lifestyle-focused educational strategies, even those not specifically targeting hypertension, are feasible and can be implemented in primary care alongside usual medication treatment. In a population frequently burdened by polypharmacy, such educational programs offer a vital pathway to achieving cardiovascular stability without increasing the pharmacological load. Future research should investigate whether the long-term adoption of these lifestyle shifts could potentially prevent the need for future escalation of antihypertensive treatments in the frail phenotype.

## Figures and Tables

**Table 1:** Descriptive Characteristics.

	Total (n = 210)		Control Group (n = 95)		Intervention Group (n = 115)		*p* Value
Age (years; mean (SD))	74.4	(6.5)	75.8	(6.8)	73.1	(6.1)	0.003
Height (cm; mean (SD))	159.0	(8.9)	162.4	(8.6)	156.7	(8.4)	<0.001
Body mass (kg; mean (SD))	73.7	(14.3)	75.2	(15.0)	72.5	(13.6)	0.176
BMI (kg/m^2^; mean (SD))	29.1	(4.8)	28.6	(4.6)	29.1	(4.9)	0.161
Systolic BP (mmHg; median [IQR])	130	[120–140]	130	[120–140]	130	[120–140]	0.172
Diastolic BP (mmHg; median [IQR])	76	[70–80]	75	[70–80]	78	[70–80]	0.215
Sex (n, %)							
Men	65	31%	42	44%	23	20%	<0.001
Women	145	69%	53	56%	92	80%	

Data is shown as mean (SD) for normally distributed continuous variables, or as median [IQR] for non-normally distributed continuous variables. Categorical variables are presented as counts (percentages). Abbreviations: SD, standard deviation; BMI, body mass index; BP, blood pressure; IQR, interquartile range.

## Data Availability

The data presented in this study are available on request from the Principal Investigator (Dr. Cristina Casals: cristina.casals@uca.es) due to ethical and privacy restrictions in accordance with the EU General Data Protection Regulation (GDPR). Anonymized data supporting the findings of this study are available upon reasonable request from qualified academic researchers, subject to the signing of a data usage agreement.

## Supplementary Materials

The following supporting information can be downloaded at: https://www.mdpi.com/article/10.3390/healthcare14060756/s1, Table S1: Mean and standard deviation of BP outcomes by group; Figure S1: Distribution of Systolic BP. Histogram showing the distribution of systolic blood pressure values (mmHg) for the control and intervention groups at the 6-month follow-up. The vertical dashed line represents the standard clinical target and diagnostic threshold for hypertension (140 mmHg); Figure S2: Distribution of Diastolic BP. Histogram showing the distribution of diastolic blood pressure values (mmHg) for the control and intervention groups at the 6-month follow-up. The vertical dashed line represents the standard clinical target and diagnostic threshold for hypertension (90 mmHg).

## Abbreviations

The following abbreviations are used in this manuscript:

BP	Blood Pressure
NTC	National Clinical Trial
CONSORT	Consolidated Standards of Reporting Trials
VREM	Spanish short version of the Minnesota Leisure Time Activity Questionnaire
ICC	Intraclass Correlation Coefficient
SPSS	Statistical Package for the Social Sciences
USA	United States of America
BMI	Body Mass Index
SD	Standard Deviation
IQR	Interquartile Range
