# Supplementary material for: Effects of a 6-Month Educational Program on Blood Pressure in Pre-Frail and Frail Older Adults: A Randomized Controlled Trial
Source: Healthcare (Basel). 2026 Mar 18;14(6):756. doi: 10.3390/healthcare14060756 (PMC13026366; doi:10.3390/healthcare14060756)

Supplementary Table S1. Mean and standard deviation of BP outcomes by group.

	Control group (n=95)		Intervention group (n=115)	
	**Baseline**	**Post-Intervention**	**Baseline**	**Post-Intervention**
Systolic BP (mmHg; mean (SD))	129.6 (14.0)	129.1 (9.5)	133.1 (17.1)	128.3 (14.7)
Diastolic BP (mmHg; mean (SD))	74.0 (9.5)	75.0 (9.8)	75.8 (10.1)	75.7 (9.3)

Data is shown as mean (Standard deviation). Abbreviations: BP, blood pressure

Supplementary Figure S1. Distribution of Systolic BP. Histogram showing the distribution of systolic blood pressure values (mmHg) for the control and intervention groups at the 6-month follow-up. The vertical dashed line represents the standard clinical target and diagnostic threshold for hypertension (140 mmHg).

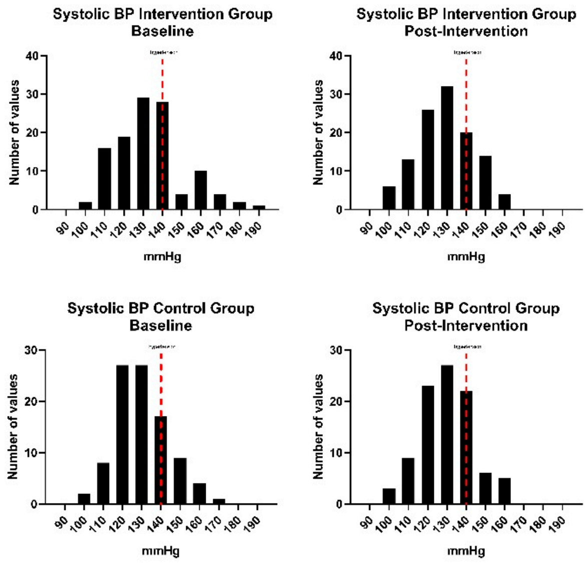

Supplementary Figure S2. Distribution of Diastolic BP. Histogram showing the distribution of diastolic blood pressure values (mmHg) for the control and intervention groups at the 6-month follow-up. The vertical dashed line represents the standard clinical target and diagnostic threshold for hypertension (90 mmHg).

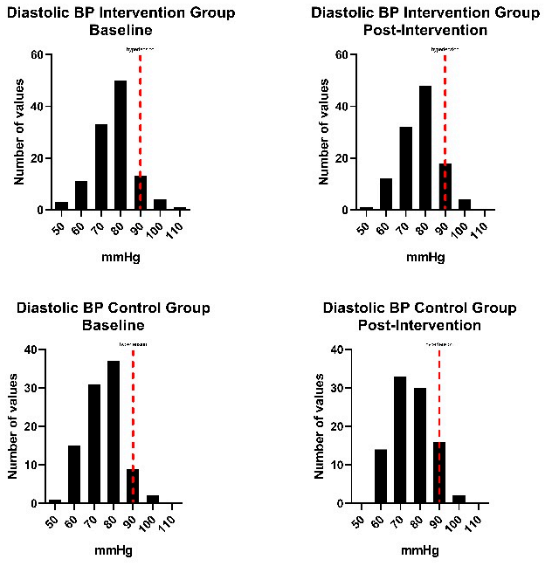

Supplement: Supplementary file 1 [file healthcare-14-00756-s001.zip › healthcare-4183991-supplementary.pdf]
